# Targeted genome engineering in human induced pluripotent stem cells from patients with hemophilia B using the CRISPR-Cas9 system

**DOI:** 10.1186/s13287-018-0839-8

**Published:** 2018-04-06

**Authors:** Cuicui Lyu, Jun Shen, Rui Wang, Haihui Gu, Jianping Zhang, Feng Xue, Xiaofan Liu, Wei Liu, Rongfeng Fu, Liyan Zhang, Huiyuan Li, Xiaobing Zhang, Tao Cheng, Renchi Yang, Lei Zhang

**Affiliations:** 1 State Key Laboratory of Experimental Hematology, Key Laboratory of Gene Therapy of Blood Diseases, Institute of Hematology and Blood Disease Hospital, Chinese Academy of Medical Sciences & Peking Union Medical College, 288 Nanjing Road, Tianjin, 300020 China; 2 Department of Hematology, The First Central Hospital of Tianjin, Tianjin, 300192 China; 3 Department of Transfusion Medicine, Shanghai Changhai Hospital, Second Military Medical University, 168 Changhai Road, Shanghai, 200433 China; 4 Division of Regenerative Medicine MC1528B, Department of Medicine, Loma Linda University, 11234 Anderson Street, Loma Linda, CA 92350 USA

**Keywords:** CRISPR-Cas systems, Induced pluripotent stem cells, Hemophilia B, Genetic therapy, Cellular therapy, Hepatocyte differentiation

## Abstract

**Background:**

Replacement therapy for hemophilia remains a lifelong treatment. Only gene therapy can cure hemophilia at a fundamental level. The clustered regularly interspaced short palindromic repeats–CRISPR associated nuclease 9 (CRISPR-Cas9) system is a versatile and convenient genome editing tool which can be applied to gene therapy for hemophilia.

**Methods:**

A patient’s induced pluripotent stem cells (iPSCs) were generated from their peripheral blood mononuclear cells (PBMNCs) using episomal vectors. The AAVS1-Cas9-sgRNA plasmid which targets the AAVS1 locus and the AAVS1-EF1α-*F9* cDNA-puromycin donor plasmid were constructed, and they were electroporated into the iPSCs. When insertion of *F9* cDNA into the AAVS1 locus was confirmed, whole genome sequencing (WGS) was carried out to detect the off-target issue. The iPSCs were then differentiated into hepatocytes, and human factor IX (hFIX) antigen and activity were measured in the culture supernatant. Finally, the hepatocytes were transplanted into non-obese diabetic/severe combined immunodeficiency disease (NOD/SCID) mice through splenic injection.

**Results:**

The patient’s iPSCs were generated from PBMNCs. Human full-length *F9* cDNA was inserted into the AAVS1 locus of iPSCs of a hemophilia B patient using the CRISPR-Cas9 system. No off-target mutations were detected by WGS. The hepatocytes differentiated from the inserted iPSCs could secrete hFIX stably and had the ability to be transplanted into the NOD/SCID mice in the short term.

**Conclusions:**

PBMNCs are good somatic cell choices for generating iPSCs from hemophilia patients. The iPSC technique is a good tool for genetic therapy for human hereditary diseases. CRISPR-Cas9 is versatile, convenient, and safe to be used in iPSCs with low off-target effects. Our research offers new approaches for clinical gene therapy for hemophilia.

**Electronic supplementary material:**

The online version of this article (10.1186/s13287-018-0839-8) contains supplementary material, which is available to authorized users.

## Background

Hemophilia, caused by mutations of coagulation factors VIII *(F8)* and IX *(F9)*, is one of the best-known hereditary hemorrhagic disorders. The current standard treatment is replacement therapy, which is effective but runs the risk of viral infection, and remains a lifelong treatment. Only gene therapy can cure hemophilia at a fundamental level [1]. The relatively small size of the *F9* coding sequence (~ 1.5 kb) makes hemophilia B (HB) a better target for genetic research compared with *F8* which causes hemophilia A (HA). Adeno-associated viral (AAV) vectors are widely used in the gene therapy for HB. A recent clinical trial indicated that HB patients retained 1–6% of the normal FIX value over 3 years after AAV8 vector injection [2], and the trial is still in progress. However, immune responses to the AAV capsid limit the wider application of this approach.

In 2013, a versatile and convenient genome editing tool, the clustered regularly interspaced short palindromic repeats–CRISPR associated nuclease 9 (CRISPR-Cas9) system, was introduced [3, 4]. The system robustly cuts DNA molecules at fixed points through Cas9 endonuclease action in the guide of an engineered single guide RNA (sgRNA). DNA double-strand breaks (DSB) produced by Cas9 can be further processed by homology-directed repair (HDR) and result in precise knock-in of exogenous genes of interest, thereby achieving overexpression of these genes. Compared with traditional gene editing tools, such as zinc finger nucleases (ZFNs) and transcription activator-like effector nucleases (TALENs), CRISPR-Cas9 is more efficient, much easier to operate, achieves a homozygous mutant, and can bring multiple mutations in different sites at the same time [5]. So far, the CRISPR-Cas9 system has been reported for genome editing in human, mice, zebra fish, yeast, and bacteria [6–9].

The induced pluripotent stem cell (iPSC) technique is a significant breakthrough in the stem cell domain in the twenty-first century [10, 11], which promotes the development of regenerative medicine. A prominent advantage of iPSCs is that they are not immunogenic when used in personalized cellular therapy. The iPSCs come from a single individual, and are sent back to the same patient after *in-vitro* genetic engineering or differentiation, avoiding the problem of immunological rejection.

The AAVS1 locus, a “safe-harbor” site, lying in the first intron of the PPP1R12C gene on human chromosome 19, has an open chromosomal region that allows the insertion of an exogenous gene [12]. Many groups targeted the AAVS1 locus using ZFN, TALEN, or CRISPR-Cas9 and described stable transgene expression [3, 13–21]. The AAVS1 locus is an excellent choice for transgene expression, so we inserted human full-length *F9* cDNA into the AAVS1 locus of iPSCs of a HB patient using CRISPR-Cas9 in this study. The edited iPSCs were then induced to hepatocytes able to secrete human FIX (hFIX), and these hepatocytes were able to be transplanted into non-obese diabetic/severe combined immunodeficiency disease (NOD/SCID) mice in the short term.

## Methods

### Generation of iPSCs of the HB patient

The patient with HB was clinically and genetically confirmed in the Institute of Hematology and Blood Disease Hospital, Chinese Academy of Medical Sciences, Tianjin, China. iPSC lines were generated from peripheral blood mononuclear cells (PBMNCs), using four episomal vectors, as described previously [22]. PBMNCs were separated from peripheral venous blood of patients by the Ficoll-Isopaque method. The reprogramming process was completed under hypoxic conditions (4% O_2_). The four episomal vectors used were supplied by Xiaobing Zhang from the Department of Medicine at Loma Linda University, CA, USA.

### Karyotype analysis

For karyotype analysis, iPSCs were harvested after 10 passages, according to a general protocol. The slides were visualized under a microscope and 20 split cells were selected randomly.

### Teratoma formation assay

iPSCs were injected intramuscularly into NOD/SCID mice. Teratomas formed within 6–8 weeks and harvested samples were fixed in 4% paraformaldehyde overnight. Paraffin sections were stained with hematoxylin and eosin (H&E) for histological determinations.

### Immunofluorescence staining

Cells were fixed with 4% paraformaldehyde for 30 min at room temperature (RT), and blocked with blocking buffer (PBS + 5% goat serum + 0.3% BSA) for 1 h at RT. Primary antibodies were incubated overnight at 4 °C. The cells were then incubated with secondary antibodies for 1 h at 37 °C. Nuclei were counterstained with 4′,6-diamidino-2-phenylindole (DAPI) for 5 min. Descriptions of the primary and secondary antibodies are presented in Additional file 1: Table S1.

### Quantitative real-time PCR

RNA was extracted using the RNeasy Mini kit (Qiagen, Dusseldorf, Germany) following the manufacturer’s instructions. RNA was reverse transcribed using a first-strand cDNA synthesis kit (Life Technologies, Carlsbad, CA, USA). Quantitative real-time PCR (qRT-PCR) analysis was performed in triplicate, using a Step-One-Plus Real-Time PCR system (Applied Biosystems, Foster City, CA, USA). The cDNA of nontransfected PBMNCs from the patient was used as negative control, while H1 embryonic stem cells were used as positive control. The primer sequences are presented in Additional file 2: Table S2.

### Construction of CRISPR-Cas9 related plasmids

An AAVS1-Cas9-sgRNA plasmid was designed to cut the human AAVS1 locus; the sgRNA sequence was 5′-GGGGCCACTAGGGACAGGAT-3′ [3]. Two donor plasmids, the AAVS1-CAG-GFP-puromycin donor plasmid and the AAVS1-EF1α-*F9* cDNA-puromycin donor plasmid, were designed to insert GFP and *F9* cDNA into the AAVS1 locus, respectively. The AAVS1-Cas9-sgRNA plasmid, the AAVS1-CAG-GFP-puromycin donor plasmid, and the AAVS1-EF1α-puromycin empty plasmid were bought. We constructed the AAVS1-EF1α-*F9* cDNA-puromycin donor plasmid by inserting human full-length *F9* cDNA into the AAVS1-EF1α-puromycin empty plasmid. Firstly, *F9* cDNA was amplified by forward primer 5′-GGGGTACCCCGCCACCATGCAGCGCGTGAACATGATC-3′ and reverse primer 5′-CGACGCGTCGTTAAGTGAGCTTTGTTTTTTCC-3′; then *F9* cDNA and the AAVS1-EF1α-puromycin empty plasmid were digested by *Mlu*I and *Kpn*I endonuclease; the digested products were then connected by the T4 ligase; and, finally, sequencing was carried out to ensure that no error occurred.

### Transfection of HEK293T cells and iPSCs

To test the efficiency of the constructed plasmids, HEK293T cells were firstly transfected using the Lipofectamine 2000 transfection reagent (Invitrogen). iPSCs were electroporated with the same plasmids, using the Lonza Amaxa Human Stem Cell Nucleofector Starter Kit and program B-016. Forward primer (F1) 5′-TTCGGGTCACCTCTCACTCC-3′ and reverse primer (R1) 5′-GGCTCCATCGTAAGCAAACC were used to detect the full inserted fragment. When insertion failed, the amplification length was 468 bp; if successful, the amplification length was 4.9 kb. Forward primer (F2) 5′-TTCCGCATTGGAGTCGCTTTA-3′ and reverse primer (R2) 5′-GTGGGCTTGTACTCGGTCATCT-3′ were used to detect the 5′ junction point of insertion. When insertion failed, nothing could be amplified; if successful, the amplification length was 1.3 kb. GAPDH was amplified as internal control for amount of genomic DNA. Forward primer 5′-ACCCACTCCTCCACCTTT-3′ and reverse primer 5′-CTCTTGTGCTCTTGCTGGG-3′ were used, and the amplification length was 283 bp. Forward primer (*F9*-F) 5′-TGCTCCTGTACTGAGGGA-3′ and reverse primer (*F9*-R) 5′-AATGATTGGGTGCTTTGA-3′ were used to detect the expression of *F9*.

### Off-target detection of CRISPR-Cas9

Potential off-target sites that differed from the sgRNA sequence by up to five nucleotides in the genome were searched using the web-based program Cas-OFFinder (http://www.rgenome.net/cas-offinder/). After insertion was detected in iPSCs, genomic DNAs extracted from the parental iPSCs and the successful inserted iPSCs were subjected to whole genome sequencing (WGS). Unique mutations of the inserted iPSCs different from those in the parental iPSCs were selected. These unique mutations were compared with the potential off-target sites to detect whether they overlapped. WGS was carried out at BGI-Shenzhen (Shenzhen, China), yielding 30× coverage. Sequencing was performed on the HiSeq X Ten platform (Illumina, Santiago, CA, USA). Raw image files were processed by Illumina pipeline for base calling with default parameters and the sequences of each individual were generated as 150-bp paired-end reads.

### Differentiation of iPSCs into hepatocytes

The differentiation of iPSCs into hepatocytes was carried out as described previously [23], with some modifications: 0.5 μM A83–01 and 0.1 μM Compound E were added during days 11–15.

### Characterization of hepatocytic functions

We detected FOXA2, SOX17, and GATA4 on day 5, hepatocyte nuclear factor 4 alpha (HNF4α) on day 10, alpha-fetoprotein (AFP) on day 15, and albumin (ALB) on day 20 by immunofluorescence staining. In addition, flow cytometry analysis was performed to detect AFP and ALB according to the provided protocols. The expressions of AFP, ALB, HNF4α, tryptophan 2,3-dioxygenase (TDO2), tyrosine-alpha-testosterone (TAT), and CYP3A4 were detected by qRT-PCR. The primer sequences are presented in Additional file 2: Table S2. For Periodic Acid–Schiff (PAS) staining, after fixation using 4% paraformaldehyde, cells were cultured in 1% periodic acid at 37 °C for 20 min, were dried at 37 °C for 1 h, and then were cultured in Schiff reagent at 37 °C for 30 min in the dark. For low-density lipoprotein (LDL) uptake and LDL-receptor expression, experiments were performed by the protocol provided by the LDL Uptake Cell-Based Assay Kit (Cayman Chemical). For indocyanine green (ICG) uptake, cells were incubated with ICG (1 mg/ml) in basal medium for 1 h at 37 °C, and then in fresh medium.

### Transplantation of hepatocytes into NOD/SCID mice

Four-week-old NOD/SCID mice were treated with retrosine (70 mg/kg, 4 weeks and 2 weeks before transplantation) and carbon tetrachloride (CCl_4_, 0.5 ml/kg, 24 h before transplantation). A total of 4.2 × 10^7^/kg hepatocytes were transplanted through splenic injection. Plasma samples were collected to detect hFIX antigen every 2 weeks after transplantation, and mice liver tissues were obtained for human ALB detection by immunohistochemistry according to general protocols.

### hFIX antigen assay

The assay of hFIX antigen in cell culture supernatant and plasma of NOD/SCID mice was carried out using the human Factor IX ELISA kit (Abcam, Cambridge, UK) according to the manufacturer’s protocol.

### hFIX activity assay

To analyze hFIX activity, Coagulation Factor IX Deficient Plasma (Siemens, Newark, DE, USA) and an Automatic Coagulation Analyzer (Sysmex CS-5100; Sysmex Kobe, Japan) were used to examine the activated partial thromboplastin time (APTT), which was compared with the calibration curve of standard plasma for calculating the hFIX content. The measurement was performed according to the manufacturer’s instructions, and a reference curve was constructed using serial dilutions of standard human plasma (Siemens).

### Statistical analysis

Data were presented as mean ± SEM and were compared by Student’s *t* test. *P* < 0.05 was considered statistically significant.

## Results

### Generation of patient-specific iPSCs from PBMNCs

**Fig. 1:**
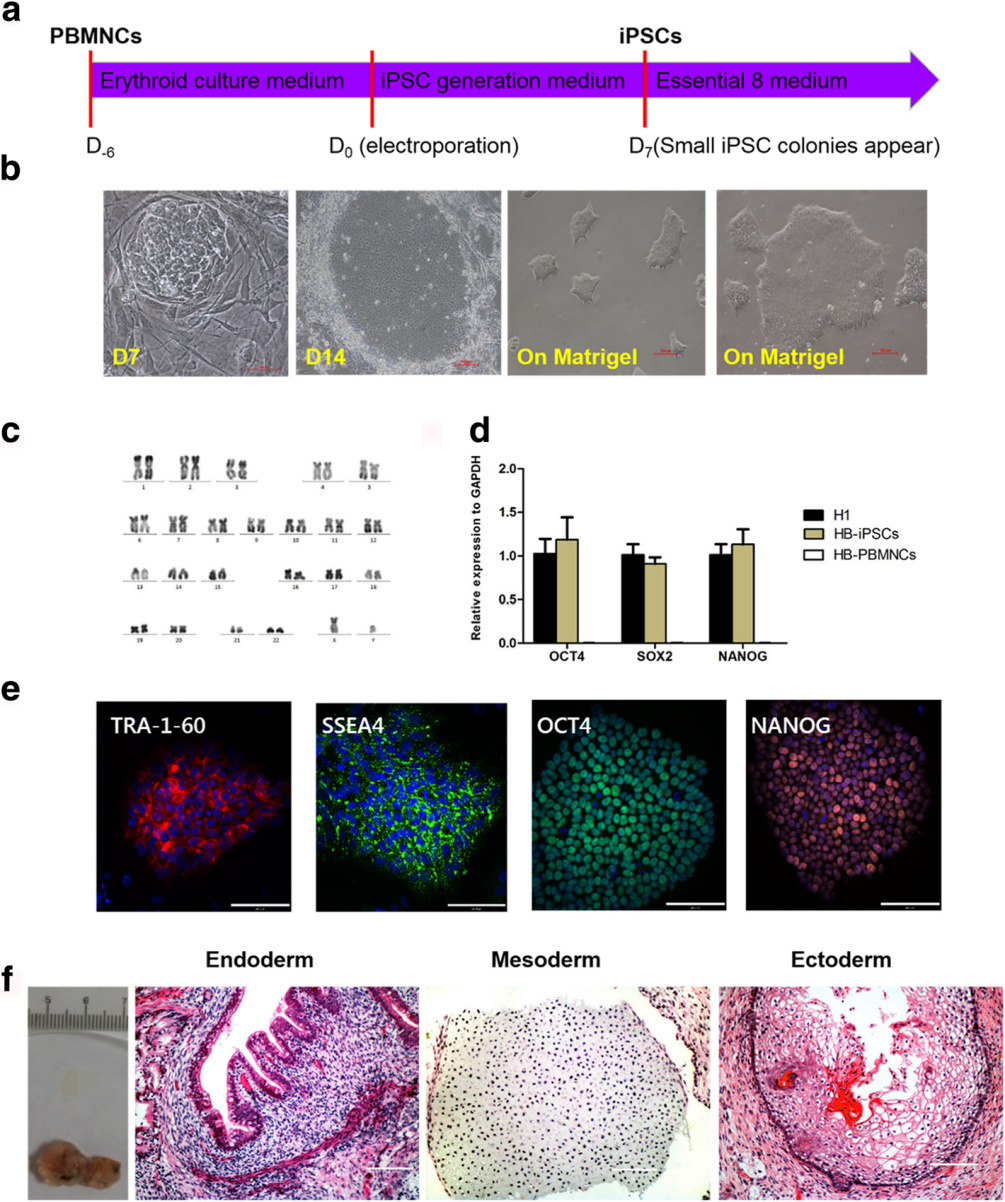
Generation and characterization of patient-specific iPSCs from PBMNCs. **a** Schematic of patient-specific iPSC generation from PBMNCs. **b** Small iPSC-like colonies appeared 7 days after electroporation. At 14 days after electroporation, iPSC colonies grew big enough for picking out. After being picked out, iPSCs were cultured on Matrigel-coated plates without feeder. **c** Karyotype of iPSCs was normal. **d** qRT-PCR analysis showed expression of OCT4, SOX2, and NANOG of iPSCs (*n* = 3 independent experiments for each sample). PBMNCs from patient used as negative control, H1 embryonic stem cells used as positive control. **e** Immunofluorescence staining showed expression of TRA-1-60, SSEA4, OCT4, and NANOG. **f** Sections of teratomas stained with H&E (endoderm: bronchus; mesoderm: cartilage; ectoderm: epidermis). All scale bars represent 100 μm. PBMNC peripheral blood mononuclear cell, iPSC induced pluripotent cell, D day, GAPDH glyceraldehyde 3-phosphate dehydrogenase, HB hemophilia B

We collected PBMNCs of an 11-year-old male HB patient from China, with a known *F9* gene mutation: c.676C > T, p.Arg226Trp. As described previously [22], isolated PBMNCs were cultured in erythroid culture medium for 6 days, and then transfected with integration-free episomal plasmids. After 7-day culture in iPSC generation medium, small iPSC colonies were observed, which were then cultured in Essential 8 medium (Fig. 1a). When iPSC colonies grew large enough, cells were removed, cultured in Essential 8 medium on Matrigel-coated plates, and passaged into small pieces using 0.5 mM EDTA (Fig. 1b). After 10 passages, karyotype and pluripotency detection were carried out. The karyotype was normal (Fig. 1c), with 22 pairs of autosomes and a pair of sex chromosomes. qRT-PCR (Fig. 1d) and immunofluorescence staining (Fig. 1e) showed expression of pluripotent markers, including OCT4, SOX2, NANOG, TRA-1-60, and SSEA4. iPSCs produced teratomas containing multiple derivatives of three germ layers after being injected into NOD/SCID mice (Fig. 1f). The iPSCs were sequenced to ensure the existence of mutation c.676C > T (Additional file 3: Figure S1a).

### Successful insertion of *F9* cDNA into the AAVS1 locus of HEK 293 T cells and HB-iPSCs

**Fig. 2:**
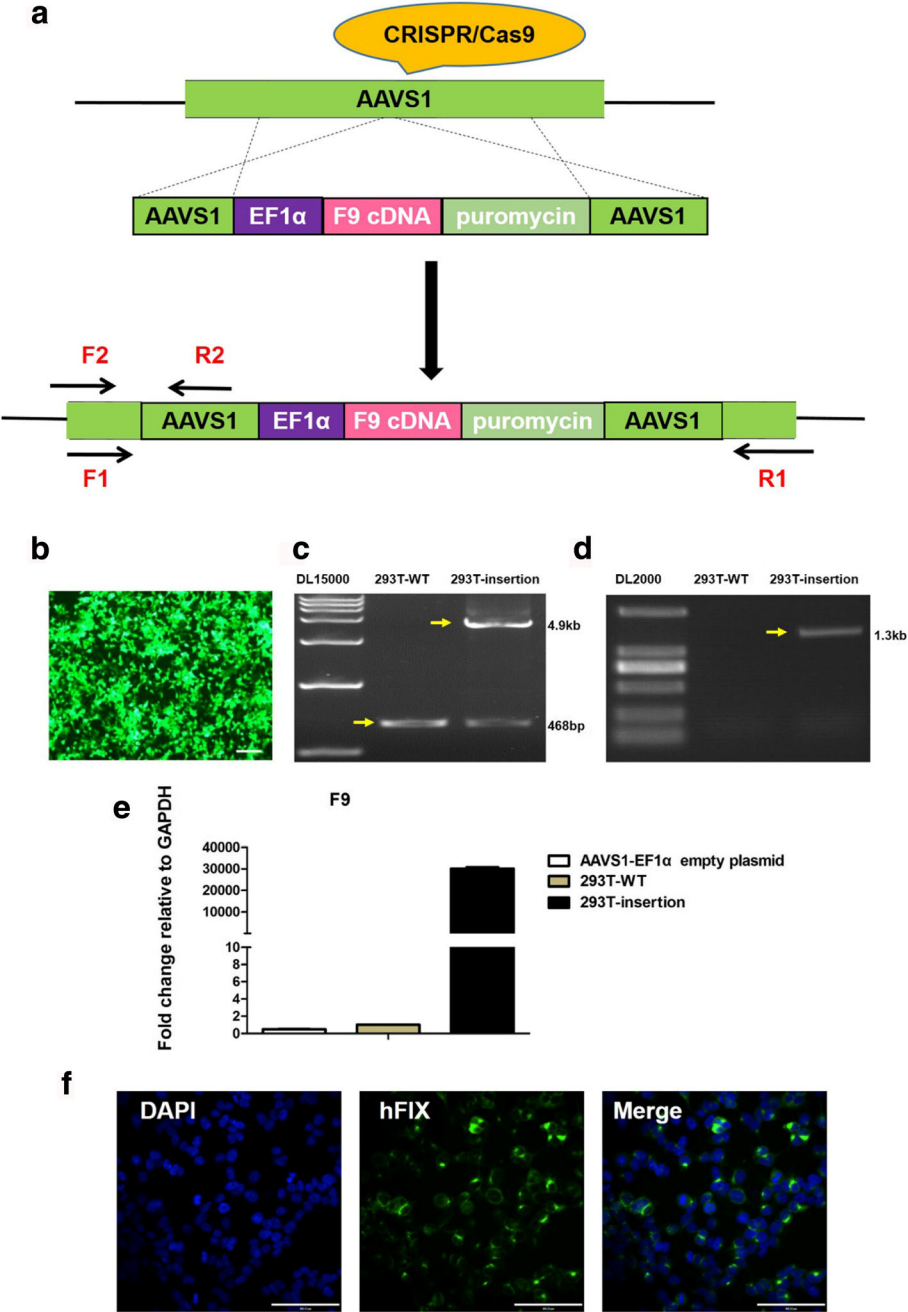
CRISPR-Cas9 and insertion of *F9* cDNA into AAVS1 locus of HEK293T cells. **a** Schematic of CRISPR-Cas9. **b** About 80% of cells were GFP-positive 24 h after transfection in the GFP group. **c** Primers F1, R1 were used to detect the full fragment of insertion. In the 293 T-WT group, only a 468-bp fragment could be seen (yellow arrow); in the 293 T-insertion group, both 468-bp and 4.9-kb fragments could be seen (yellow arrow). **d.** Primers F2, R2 used to detect 5′ junction point of insertion. In the 293 T-WT group, nothing could be seen; in the 293 T-insertion group, a 1.3-kb fragment could be seen (yellow arrow). **e** qRT-PCR showed *F9* expression of the 293 T-insertion group was extremely higher than that in the 293 T-WT group. AAVS1-EF1α-puromycin empty plasmid did not produce any hFIX expression (n = 3 independent experiments for each sample). **f** Immunofluorescence staining showed hFIX expression in the 293 T-insertion group. All scale bars represent 100 μm. CRISPR-Cas9 clustered regularly interspaced short palindromic repeats-CRISPR associated nuclease 9, WT wildtype, GAPDH glyceraldehyde 3-phosphate dehydrogenase, DAPI 4′,6-diamidino-2-phenylindole, HFIX human factor IX

In this study, we attempted to insert human full-length *F9* cDNA into the AAVS1 locus to acquire stable expression of hFIX. Fig. 2a shows the schematic diagram of CRISPR-Cas9. Firstly, HEK 293 T cells were transfected to detect the efficiency of the plasmids. Additional file 4: Table S3 presents the dosages of plasmids and cell numbers used in the study. We observed that approximately 80% of cells were GFP-positive 24 h after transfection in the GFP group (Fig. 2b). Forty-eight hours after transfection, 2 μg/ml puromycin was used for drug selection in both groups. After three passages, the remaining cells were assayed for *F9* expression in the *F9* group. Insertion could be detected using primers F1, R1 and F2, R2 in the 293 T-insertion group (Fig. 2c, d). The expression of *F9* in the 293 T-insertion group was notably higher than that in the 293 T-wildtype (WT) group (Fig. 2e). hFIX was also detected in the 293 T-insertion group through immunofluorescence staining (Fig. 2f). Collectively, these results demonstrated the efficiency of the plasmids.

**Fig. 3:**
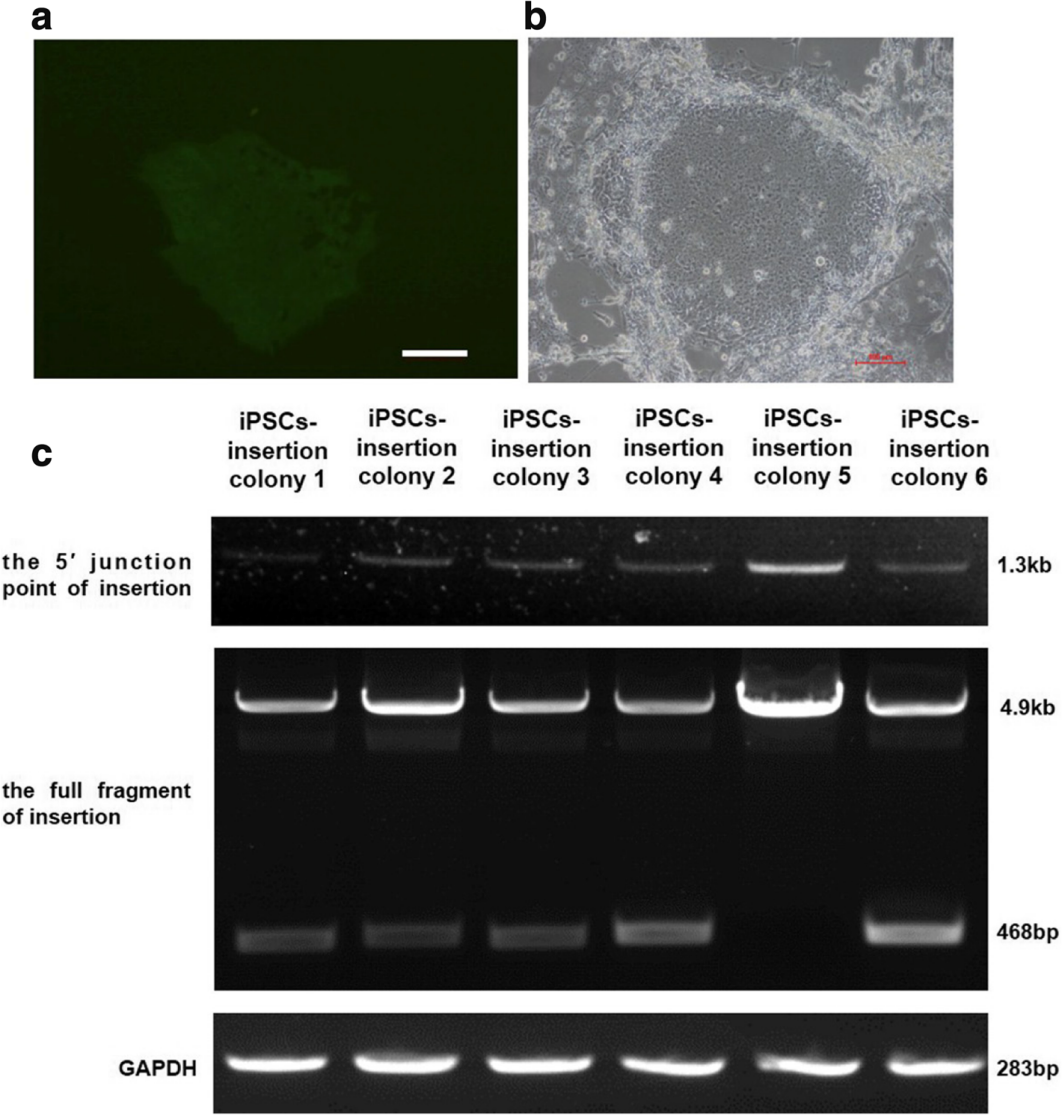
Insertion of *F9* cDNA into AAVS1 locus of HB iPSCs. **a** Surviving cells grew large in the GFP group after puromycin selection. **b** Surviving cells (colony 5) grew large in the *F9* group after puromycin selection. **c** Primers F2, R2 used to detect 5′ junction point of insertion. A 1.3-kb fragment could be seen in all iPSC colonies; primers F1, R1 used to detect the full fragment of insertion. The 468-bp and 4.9-kb fragments could be seen in iPSC colonies 1, 2, 3, 4 and 6; while only a 4.9-kb fragment could be seen in iPSC colony 5, there was no 468-bp fragment. GAPDH was amplified as internal control of amount of genomic DNA. A 283-bp fragment of same intensity could be seen in all iPSC colonies. All scale bars represent 100 μm. iPSC induced pluripotent cell, GAPDH glyceraldehyde 3-phosphate dehydrogenase

To transfect HB-iPSCs, 1 × 10^6^ iPSCs were treated with 10 μM ROCK inhibitor Y26732 for 4–6 h. After electroporation, the iPSCs were seeded onto feeder cells. Twenty-four hours later, sporadic GFP-positive cells could be seen in the GFP group. Forty-eight hours after transfection, 0.3 μg/ml puromycin was used for drug selection. Most iPSCs died after drug selection, but a few survived. After about 7 days, each surviving iPSC colony grew large enough for picking out in both groups (Fig. 3a, b) for further insertion detection. We picked out six colonies in total in the *F9 *group. As shown in Fig. 3c, using primers F2 and R2, a 1.3-kb fragment could be detected in all iPSC colonies, indicating the successful 5′ junction; using primers F1 and R1, 468-bp and 4.9-kb fragments could be seen in iPSC colonies 1, 2, 3, 4, and 6, indicating heterozygous insertion of *F9* cDNA; only a 4.9-kb fragment could be seen in iPSC colony 5, indicating homozygous insertion of *F9* cDNA. Additional file 3: Figure S1b shows the sequencing result of *F9* mutation (c.676C > T) of colony 5. The karyotype of iPSC colony 5 was normal and its pluripotency was well detected (Additional file 5: Figure S2). So, iPSC colony 5 (referred to as iPSC-insertion) was used for further hepatocytic differentiation.

### Off-target effect detection in the successfully inserted iPSCs

Using Cas-OFFinder, 1799 potential off-target sites that differed from the sgRNA sequence by up to five nucleotides in the genome were found. We found 97,968 indels, 3084 structural variations (SVs), 51,628 single nucleotide polymorphisms (SNPs), and 2225 copy number variations (CNVs) unique to the inserted iPSCs compared to that in the parental iPSCs. Since indels and SVs comprise virtually all of the mutations introduced by CRISPR-Cas9 [24], we focused solely on indels and SVs. Through comparison of potential off-target sites, and indels and SVs unique to the inserted iPSCs, we did not find any overlapping mutation between them (Additional file 6: Figure S3).

### Functional hepatocyte differentiation of inserted iPSCs

**Fig. 4:**
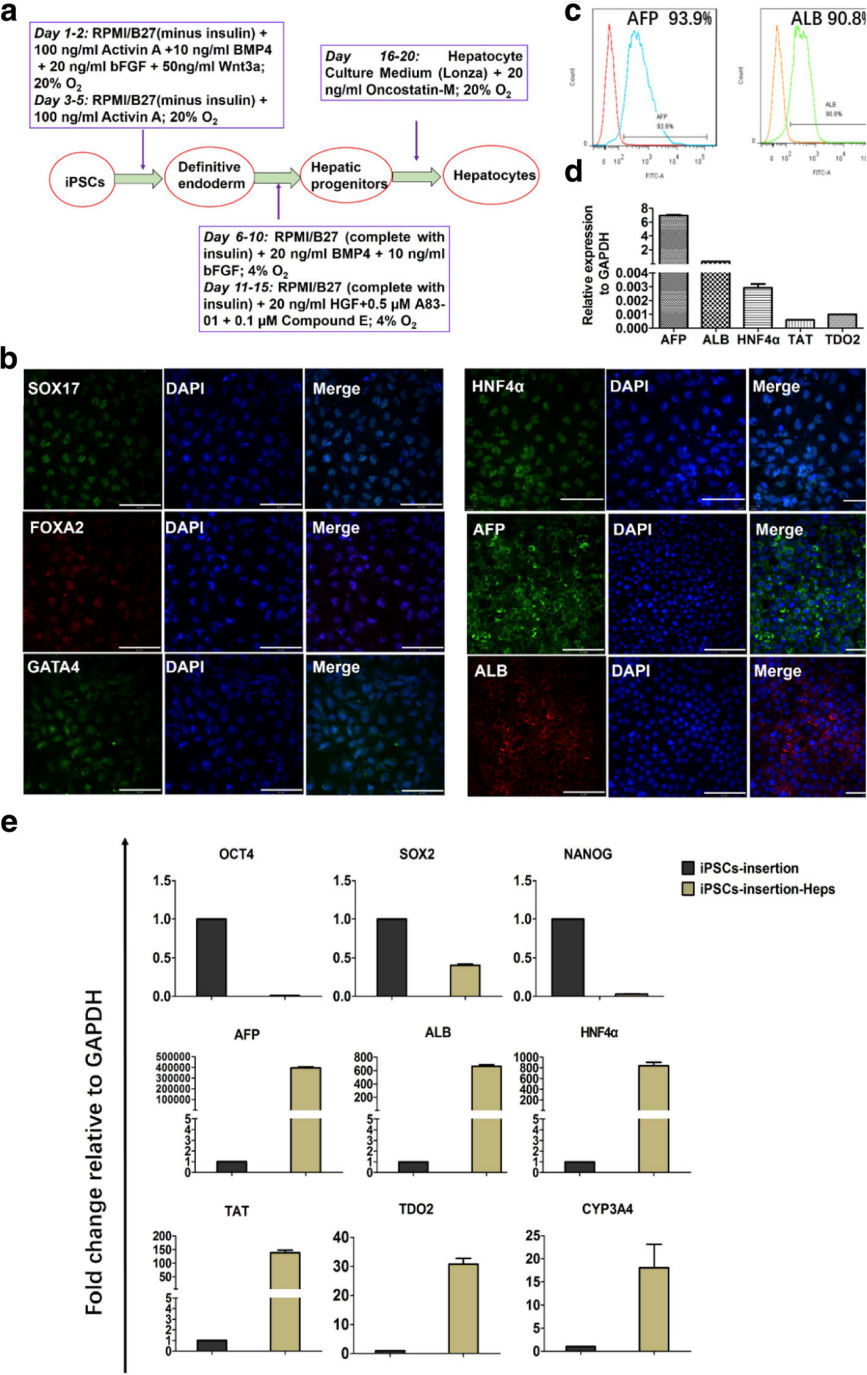
Differentiation of iPSCs into hepatocytes and characterization of hepatocytic functions. **a** Schematic showing stepwise protocol for producing hepatocytes from iPSCs. **b** Immunofluorescence staining showed expression of SOX17, FOXA2, and GATA4 on day 5, HNF4α on day 10, AFP on day 15, and ALB on day 20. **c** Flow cytometry analysis showed expression of AFP and ALB > 90%. **d** qRT-PCR showed relative expression of HNF4α, AFP, ALB, TDO2, and TAT relative to GAPDH (n = 3 independent experiments for each sample). **e** Expression of pluripotent markers (OCT4, SOX2, and NANOG) and hepatic markers (AFP, ALB, HNF4α, TAT, TDO2, and CYP3A4) between iPSC-insertion and iPSC-insertion-Heps compared by qRT-PCR (n = 3 independent experiments for each sample). After differentiation, expression of pluripotent markers decreased, while expression of hepatic markers increased. All scale bars represent 100 μm. BMP bone morphogenetic protein, bFGF basic fibroblast growth factor, iPSC induced pluripotent cell, HGF, hepatocyte growth factor, DAPI 4′,6-diamidino-2-phenylindole, AFP alpha-fetoprotein, ALB albumin, GAPDH glyceraldehyde 3-phosphate dehydrogenase

When iPSCs acquired a confluence of 50–70%, the differentiation was performed as shown in Fig. 4a. The total differentiation process took 20 days, with days 6–15 under 4% O_2_. Immunofluorescence staining indicated expression of FOXA2, SOX17, and GATA4 on day 5, HNF4α on day 10, AFP on day 15, and ALB on day 20 (Fig. 4b). Flow cytometry analysis indicated that the expression of AFP and ALB was greater than 90% (Fig. 4c). Then we detected the hepatic markers at the gene level. qRT-PCR showed the expression of AFP, ALB, HNF4α, TDO2, and TAT relative to GAPDH (Fig. 4d). It was interesting to note that the marker of precursor hepatocytes (AFP) was high, whereas the markers of mature hepatocytes (ALB, TDO2, and TAT) were relatively low, indicating that the differentiated hepatocytes we obtained were still in the process of further maturing. Compared to the expression of ALB in flow cytometry (Fig. 4c), the ALB expression in qRT-PCR (Fig. 4d) was relatively low, which might be caused by the asynchrony of gene transcription and protein translation. Then we compared the expression of pluripotent markers (OCT4, SOX2, and NANOG) and hepatic markers (AFP, ALB, HNF4α, TDO2, TAT, and CYP3A4) between the undifferentiated iPSCs and the differentiated hepatocytes (referred to as iPSC-insertion-Heps) by qRT-PCR (Fig. 4e). After differentiation, the expression of pluripotent markers decreased, whereas the expression of hepatic markers increased. The differentiated cells had the function of glycogen storage (Additional file 7: Figure S4a) and ICG uptake (Additional file 7: Figure S4b). The differentiated cells also expressed LDL-receptor (Additional file 7: Figure S4c) and had the ability for LDL uptake (Additional file 7: Figure S4d).

### *F9* expression, and hFIX antigen levels and activity in the supernatant medium

**Fig. 5:**
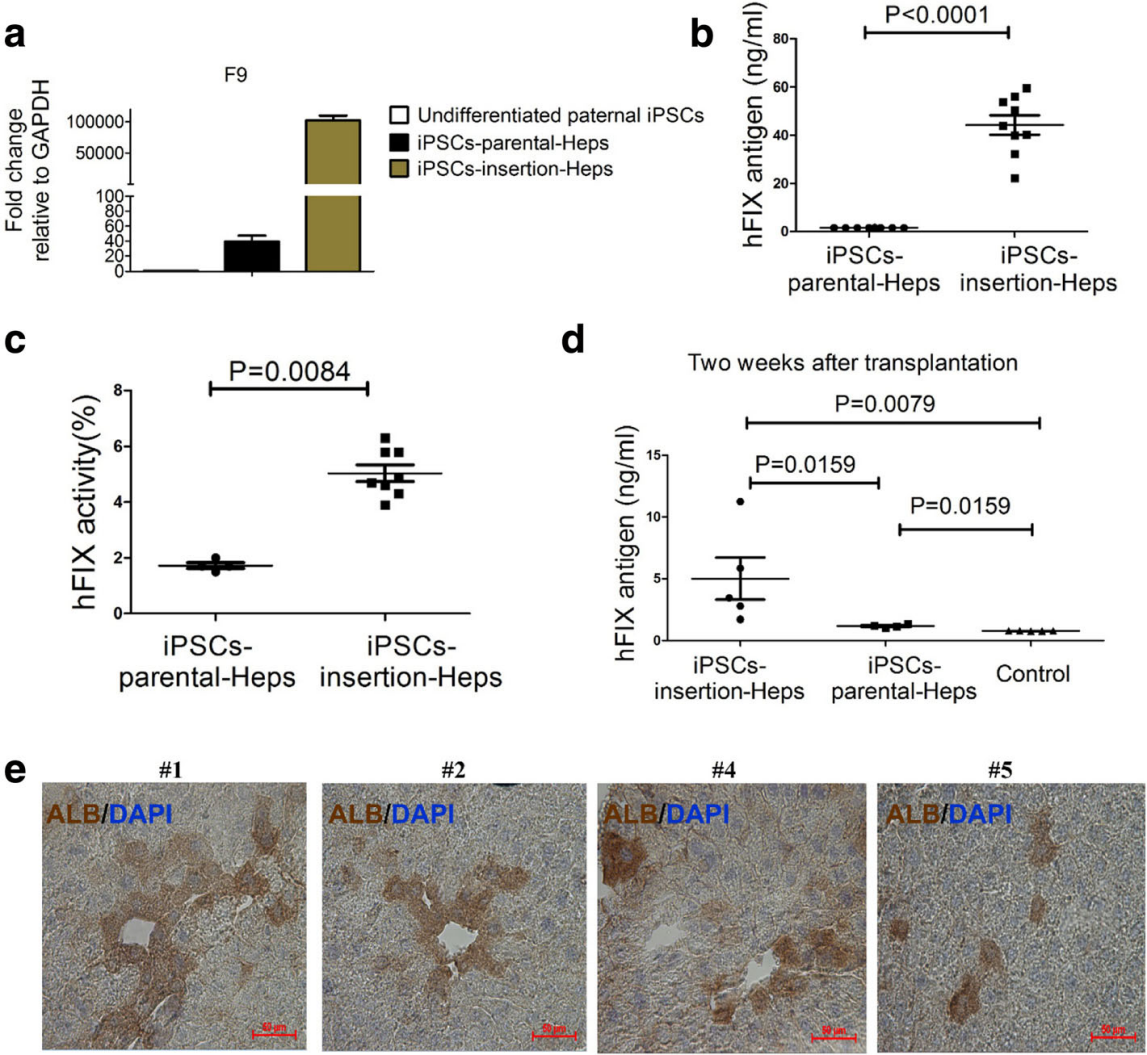
Comparison of *F9* expression, and hFIX antigen levels and activity between iPSC-parental-Heps and iPSC-insertion-Heps. **a** qRT-PCR showed *F9* expression in undifferentiated iPSCs, iPSC-parental-Heps, and iPSC-insertion-Heps (n = 3 independent experiments for each sample). **b** Comparison of hFIX antigen levels in cell culture supernatant between iPSC-parental-Heps and iPSC-insertion-Heps. Student’s t test used. Data shown are mean ± SEM (*n* = 8 in iPSC-parental-Heps group, *n* = 9 in iPSC-insertion-Heps group). **c** Comparison of hFIX activity in cell culture supernatant between iPSC-parental-Heps and iPSC-insertion-Heps. Student’s t test used. Data shown are mean ± SEM (*n* = 4 in iPSC-parental-Heps group, n = 8 in iPSC-insertion-Heps group). **d** Two weeks after transplantation, hFIX antigen levels in mice plasma of the iPSC-insertion-Heps group, iPSC-parental-Heps group, and control group detected by ELISA. Student’s t test used. Data shown are mean ± SEM (*n* = 5 in iPSC-insertion-Heps group and the control group, n = 4 in iPSC-parental-Heps group). **e** Two weeks after transplantation, human ALB was detected in mice #1, #2, #4, and #5 of the iPSC-insertion-Heps group by immunohistochemistry. All scale bars represent 50 μm. GAPDH glyceraldehyde 3-phosphate dehydrogenase, iPSC induced pluripotent cell, hFIX human factor IX, DAPI 4′,6-diamidino-2-phenylindole, ALB albumin

We compared *F9* expression between iPSC-insertion-Heps and differentiated hepatocytes from the patient’s uninserted iPSCs (referred to as iPSC-parental-Heps) by qRT-PCR (Fig. 5a). qRT-PCR indicated that *F9* expression in iPSC-insertion-Heps was notably higher than that in the other two groups. hFIX antigen in cell culture supernatant of iPSC-insertion-Heps was higher than that in iPSC-parental-Heps (44.357 ± 4.035 ng/ml vs 1.575 ± 0.004 ng/ml, *P* < 0.0001) (Fig. 5b) at the end of the 20th day. hFIX activity in cell culture supernatant of iPSC-insertion-Heps was also higher than that in iPSC-parental-Heps (5.038 ± 0.296% vs 1.725 ± 0.103%, *P* = 0.0084) (Fig. 5c).

### Implanting ability of the differentiated hepatocytes

Fifteen NOD/SCID mice were divided randomly into three equal groups. iPSC-insertion-Heps were injected into the first group, iPSC-parental-Heps were injected into the second group, and 100 μl basic Hepatocyte Culture medium (Lonza) was injected into the third group as control. Two weeks after transplantation, only one mouse in the iPSC-parental-Heps group died, and all of the others survived. We detected hFIX antigen in mice plasma by ELISA (Fig. 5d). hFIX antigen levels in the iPSC-insertion-Heps group was higher than that in the iPSC-parental-Heps group (4.998 ± 1.703 ng/ml vs 1.178 ± 0.076 ng/ml, *P* = 0.0159) and in the control group (4.998 ± 3.808 ng/ml vs 0.789 ± 0.003 ng/ml, *P* = 0.0079). hFIX antigen levels in the iPSC-parental-Heps group was higher than that in the control group (1.178 ± 0.076 ng/ml vs 0.789 ± 0.003 ng/ml, *P* = 0.0159). We then detected human ALB in four mice of the iPSC-insertion-Heps group by immunohistochemistry (Fig. 5e).

## Discussion

iPSCs possess self-renewal ability and multilineage differentiation potential similar to embryonic stem cells, and are not constrained by traditional ethical issues, providing potentially revolutionary approaches in personalized cell therapy, disease modeling, and drug screening [25, 26]. Conventionally, human iPSCs were generated from skin fibroblasts. Being a hemorrhagic disease, hemophilia is not suitable for this invasive operation. As a result, previous studies generated iPSCs of hemophilia patients from urine cells [27–29]. PBMNCs have been reported as the best cell source for cell reprogramming owing to their better quality, quantity, and easy accessibility [30–34]. Therefore, we generated iPSCs of HB patients from PBMNCs in this study.

The development of genetic engineering tools opens up many opportunities for the treatment of human hereditary diseases. Notably, the CRISPR-Cas9 system is the most powerful homologous recombination-based gene editing method, and has been used in human studies of beta-thalassemia, cystic fibrosis, and sickle cell disease [35–38]. CRISPR-Cas9 has been used in hemophilia to correct structural variations and chromosomal inversions of HA patients [29, 39] and somatic mutations of HB mice [40]. A previous study suggested that GC-rich sgRNAs improved targeting efficiency, whereas poly(U) stretches close to the protospacer-adjacent motif (PAM) sequence were associated with sgRNAs of lower efficiency [41]. When designing sgRNAs for *in-situ* correction, one has to constrain the sgRNA sequences around the mutant nucleotides, which runs the risk of constructing sgRNAs with lower targeting efficiency. In this study, we used a sgRNA with high efficiency to target the AAVS1 locus and a donor plasmid carrying human full-length *F9* cDNA, and then these plasmids could be applied to all HB patients regardless of their mutation type, eliminating the trouble of constructing sgRNAs with lower targeting efficiency. This approach is particularly suitable for HA, as there are already 2015 different mutations documented in the Factor VIII Gene (F8) Variant Database (http://www.factorviii-db.org/) [42], which makes it quite difficult to generate sgRNAs with high efficiency when performing *in-situ* correction. Thus, in our future studies we will aim to knock-in human full-length *F8* cDNA or B domain-deleted (BDD) *F8* cDNA into the AAVS1 locus of iPSCs from HA patients.

Before transfecting the iPSCs, HEK293T cells were transfected firstly to test the knock-in efficiency of plasmids. HEK293T cells are used to check the cutting efficiency of sgRNAs generally [43], because HEK293T cells are easy to transfect. But the epigenome state and genome sequences of HEK293T cells are different from primary hepatocytes. High efficiency of sgRNAs in HEK293T cells does not mean high efficiency in primary hepatocytes. So primary hepatocytes with *F9* mutation or a *F9* knockout transformed cell line were better choices to detect the effect of plasmids in our further study.

A problem that cannot be ignored in CRISPR-Cas9 is off-target effects. Previous studies have shown that mismatches with 20 nucleotides of sgRNAs can be tolerated, leading to genome editing at undesired sites [44–47]. However, the off-target incidence is lower in pluripotent stem cells (PSCs) [24, 29, 43], which is consistent with lower knock-out and knock-in efficiency of sgRNAs in PSCs [48]. CRISPR-Cas9 can induce mutations at sites that differ by as many as five nucleotides from the intended 20 nucleotides [44]. Therefore, in this study, we compared potential off-target sites that differed from the sgRNA sequence by up to five nucleotides in the genome with indels and SVs unique to the inserted iPSCs, and found no overlapping mutations. As a result, no off-target mutations were found in our study.

In our research, hepatocytes differentiated from human iPSCs should be transplanted to immunodeficient mice or hemophilia mice treated with immune-suppression drugs. Since there are no immunodeficient hemophilic mice models available, we transplanted the differentiated hepatocytes into NOD/SCID mice instead. The Automatic Coagulation Analyzer (Sysmex CS-5100) we used to detect the hFIX activity could not distinguish between human FIX and mouse FIX, so we did not detect hFIX activity, but instead assayed the hFIX antigen by ELISA. hFIX antigen could be detected only 2 weeks after transplantation, and could no longer be detected after 4 weeks or later in all the groups. It was possible that newly regenerated mice hepatocytes rejected the transplanted human hepatocytes. Better ways to improve implanting efficiency should be further explored. Considering that human and NOD/SCID mice are two different species, human cells cannot transplant into NOD/SCID mice thoroughly. So it may make sense that the FIX levels in mice plasma seem to be much lower in our research. However, the situation may be completely different when human cells are retransfused into the human body where they came from, because there is no immunological rejection.

## Conclusions

We generated HB patient-specific iPSCs from PBMNCs with normal karyotype and good pluripotency. PBMNCs are better somatic cell choices for generating iPSCs from hemophilia patients. Although the efficiency of CRISPR-Cas9 in PSCs is relatively low, we successfully knocked the human full-length *F9* cDNA into the AAVS1 locus of the iPSCs without off-target effects. Therefore, it is of far-reaching significance to construct sgRNAs with high efficiency, especially when they are used in PSCs. In addition, CRISPR-Cas9 is relatively safe to be used in PSCs, considering the low off-target effects. The differentiated hepatocytes from inserted iPSCs could secrete hFIX stably and were able to be transplanted into the NOD/SCID mice in the short term, which showed great promise for further exploration of *in-vivo* research. In summary, by combining the iPSC technique with the CRISPR-Cas9 system, we provide a new approach for clinical gene therapy for hemophilia.

## Additional files

Additional file 1: **Table S1.** presenting antibodies used for immunofluorescence staining and flow cytometry analysis. (DOCX 14 kb)

Additional file 2: **Table S2.** presenting primers used for characterization of hepatocytic functions. (DOCX 14 kb)

Additional file 3: **Figure S1.** showing sequencing results of parental and inserted iPSCs. **a** Parental iPSCs have the known *F9* gene mutation c.676C > T, p.Arg226Trp. **b** Inserted iPSCs (colony 5) have a heterozygous mutation of c.676C > T, p.Arg226Trp. (DOCX 393 kb)

Additional file 4: **Table S3** presenting plasmids used for transfection of HEK293T cells and iPSCs. (DOCX 14 kb)

Additional file 5: **Figure S2** showing characterization of iPSC colony 5. **a** Karyotype of iPSC colony 5 was normal. **b** qRT-PCR analysis showed expression of OCT4, SOX2, and NANOG of iPSC colony 5. PBMNCs of patient used as negative control, H1 embryonic stem cells used as positive control. **c** Immunofluorescence staining showed expression of TRA-1-60, SSEA4, OCT4, and NANOG. **d** Sections of teratomas stained with H&E (endoderm: pancreas; mesoderm: muscle; ectoderm: nerve fibers). All scale bars represent 100 μm. (DOCX 1261 kb)

Additional file 6: **Figure S3.** showing off-target effects detection in successful inserted iPSCs. Using Cas-OFFinder, 1799 potential off-target sites that differed from the sgRNA sequence by up to five nucleotides in the genome were found. We found 97,968 indels, 3084 SVs, 51,628 SNPs, and 2225 CNVs unique to the inserted iPSCs compared to that in the parental iPSCs. Since indels and SVs comprise virtually all of the mutations introduced by CRISPR-Cas9, we focused solely on indels and SVs. Through comparison of potential off-target sites, and indels and SVs unique to the inserted iPSCs, we found no overlapping mutation between them. (DOCX 302 kb)

Additional file 7: **Figure S4.** showing characterization of hepatocytic functions. Differentiated cells had functions of glycogen storage (**a**) and ICG uptake (**b**), and also expressed LDL-receptor (**c**) and had ability for LDL uptake (**d**). All scale bars represent 100 μm. (DOCX 1747 kb)

## Abbreviations

AFP: Alpha-fetoprotein;
ALB: Albumin;
APTT: Activated partial thrombo-plastin time;
CCl_4_: Carbon tetrachloride;
CNVs: Copy number variations;
CRISPR-Cas9: Clustered regularly interspaced short palindromic repeats-–CRISPR associated nuclease 9;
DAPI: 4′ 6-Ddiamidino-2-phenylindole;
DSB: DNA double-strand breaks;
H&E: Hematoxylin and eosin;
HA: Hemophilia A;
HB: Hemophilia B;
HDR: Homology-directed repair;
ICG: Indocyanine green;
iPSC: Induced pluripotent stem cell;
LDL: Low-density lipoprotein;
NOD/SCID: Non-obese diabetic/severe combined immunodeficiency disease;
PAM: Protospacer-adjacent motif;
PAS: Periodic Acid-–Schiff;
PBMNCs: Peripheral blood mononuclear cells;
PSCs: Pluripotent stem cells;
qRT-PCR: Quantitative real-time PCR;
sgRNA: Single guide RNA;
SNPs: Single nucleotide polymorphisms;
SVs: Structural variations;
TALENs: Transcription activator-like effector nucleases;
TAT: Tyrosine- alpha-testosterone;
TDO2: Tryptophan 2,3-dioxygenase;
ZFNs: Zinc finger nucleases
