# Supplementary material for: Targeted genome engineering in human induced pluripotent stem cells from patients with hemophilia B using the CRISPR-Cas9 system
Source: Stem Cell Res Ther. 2018 Apr 6;9:92. doi: 10.1186/s13287-018-0839-8 (PMC5889534; doi:10.1186/s13287-018-0839-8)
Supplement: Supplementary file 1 — Table S1. presenting antibodies used for immunofluorescence staining and flow cytometry analysis. (DOCX 14 kb) [file 13287_2018_839_MOESM1_ESM.docx]

**Additional file 1: Table S1.** Antibodies used for immunofluorescence staining and flow cytometry analysis.

| **Antigen** | **Distributor** | **Host** | **Cat#** | **Dilution** | |
| --- | --- | --- | --- | --- | --- |
| FOXA2 | R&D | Goat | AF2400 | | 10ug/ml |
| SOX17 | R&D | Mouse | MAB1924 | | 10ug/ml |
| GATA4 | R&D | Goat | AF2606 | | 10ug/ml |
| HNF4α | Santa Cruz | Goat | sc-6556 | | 1:50 |
| AFP | Sigma | Mouse | A8452 | | 1:500 |
| ALB | R&D | Mouse | MAB1455 | | 10ug/ml |
| FIX | Santa Cruz | Mouse | sc-73457 | | 1:50 |
| Alexa Fluor® 555,Anti-Mouse | Invitrogen | Goat | A21425 | | 1:500 |
| Alexa Fluor® 488,Anti-Mouse | Invitrogen | Donkey | A21202 | | 1:500 |
| Alexa Fluor® 488,Anti-Goat | Invitrogen | Donkey | A11055 | | 1:500 |
| Alexa Fluor® 488-human AFP | R&D | - | IC1368G | | 5μl/10^6^ cells |
| Alexa Fluor® 488-human ALB | R&D | - | IC1455G | | 5μl/10^6^ cells |
