# Supplementary material for: Targeted genome engineering in human induced pluripotent stem cells from patients with hemophilia B using the CRISPR-Cas9 system
Source: Stem Cell Res Ther. 2018 Apr 6;9:92. doi: 10.1186/s13287-018-0839-8 (PMC5889534; doi:10.1186/s13287-018-0839-8)
Supplement: Supplementary file 2 — Table S2. presenting primers used for characterization of hepatocytic functions. (DOCX 14 kb) [file 13287_2018_839_MOESM2_ESM.docx]

**Additional file 2: Table S2.** Primers used for characterization of hepatocytic functions.

| Gene Symbol | Sequence（5′→3′） |
| --- | --- |
| OCT4 | F: GACAACAATGAAAATCTTCAGGAGA |
|  | R: TTCTGGCGCCGGTTACAGAACCA |
| SOX2 | F: AGCTACAGCATGATGCAGGA |
|  | R: GGTCATGGAGTTGTACTGCA |
| NANOG | F: TGAACCTCAGCTACAAACAG |
|  | R: TGGTGGTAGGAAGAGTAAAG |
| HNF4α | F: CCAAGTACATCCCAGCTTTC |
|  | R: TTGGCATCTGGGTCAAAG |
| AFP | F: GCAGCCAAAGTGAAGAGG |
|  | R: TGTTGCTGCCTTTGTTTG |
| ALB | F: CGCTATTAGTTCGTTACACCA |
|  | R: TTTACAACATTTGCTGCCCA |
| TDO2 | F: GACGGCTGTCATACAGAGCA |
|  | R: CGCAGGTAGTGATAGCCTGA |
| TAT | F: ATCTCTGTTATGGGGCGTTG |
|  | R: TGATGACCACTCGGATGAAA |
| CYP3A4 | F: GATGGCTCTCATCCCAGACTT |
|  | R: AGTCCATGTGAATGGGTTCC |
| GAPDH | F: GAGTCCACTGGCGTCTTC |
|  | R: GACTGTGGTCATGAGTCCTTC |
