# Supplementary material for: Targeted genome engineering in human induced pluripotent stem cells from patients with hemophilia B using the CRISPR-Cas9 system
Source: Stem Cell Res Ther. 2018 Apr 6;9:92. doi: 10.1186/s13287-018-0839-8 (PMC5889534; doi:10.1186/s13287-018-0839-8)
Supplement: Supplementary file 3 — Figure S1. showing sequencing results of parental and inserted iPSCs. a Parental iPSCs have the known F9 gene mutation c.676C > T, p.Arg226Trp. b Inserted iPSCs (colony 5) have a heterozygous mutation of c.676C > T, p.Arg226Trp. (DOCX 393 kb) [file 13287_2018_839_MOESM3_ESM.docx]

**
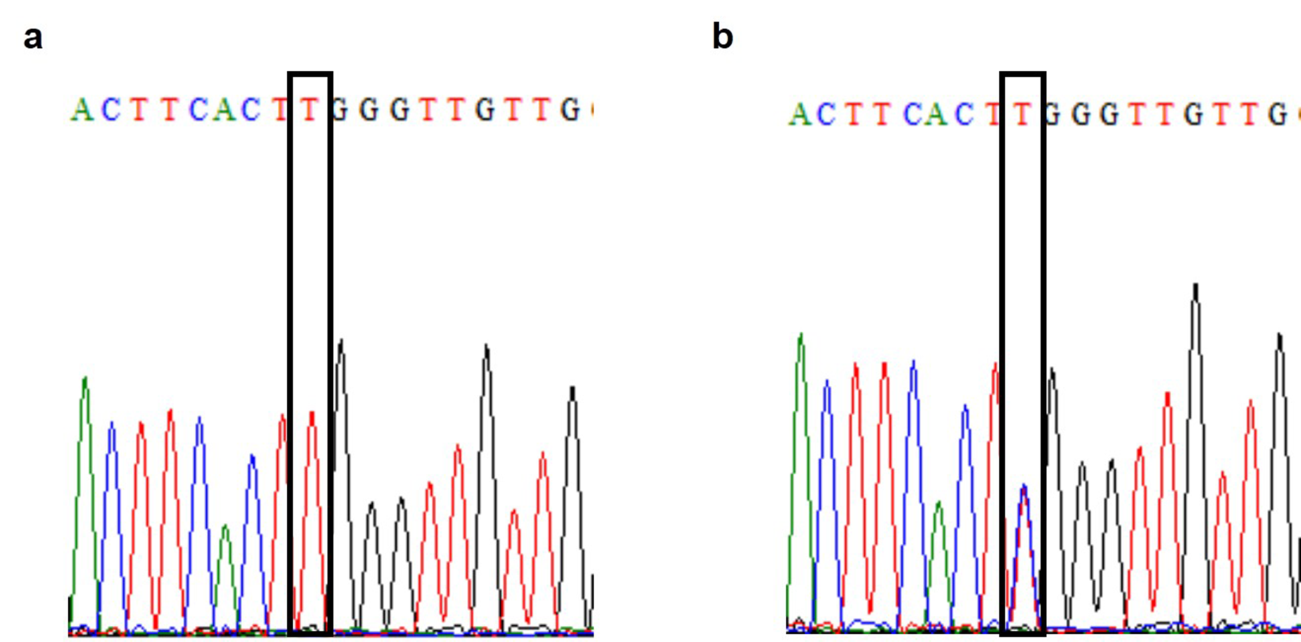
Additional file 3: Figure S1.** The sequencing results of the parental and inserted iPSCs. **a.** The parental iPSCs has a known mutation of *F9* gene: c.676C>T, p.Arg226Trp. **b.** The inserted iPSCs (colony 5) has a heterozygous mutation of c.676C>T, p.Arg226Trp.
