# Supplementary material for: Targeted genome engineering in human induced pluripotent stem cells from patients with hemophilia B using the CRISPR-Cas9 system
Source: Stem Cell Res Ther. 2018 Apr 6;9:92. doi: 10.1186/s13287-018-0839-8 (PMC5889534; doi:10.1186/s13287-018-0839-8)
Supplement: Supplementary file 4 — Table S3 presenting plasmids used for transfection of HEK293T cells and iPSCs. (DOCX 14 kb) [file 13287_2018_839_MOESM4_ESM.docx]

**Additional file 4: Table S3.** Plasmids used for transfection of HEK293T cells and iPSCs.

| Plasmids (μg) |  | HEK293T cells (4.5×10^5^) | |  | iPSCs (1×10^6^) | |
| --- | --- | --- | --- | --- | --- | --- |
|  |  | GFP group | F9 group |  | GFP group | F9 group |
| AAVS1-Cas9-sgRNA |  | 0.33 | 0.33 |  | 1.5 | 1.5 |
| AAVS1-CAG-GFP-puromycin donor |  | 0.66 |  |  | 3 |  |
| AAVS1-EF1α-F9 cDNA-puromycin donor |  |  | 0.66 |  |  | 3 |
