# Supplementary material for: Targeted genome engineering in human induced pluripotent stem cells from patients with hemophilia B using the CRISPR-Cas9 system
Source: Stem Cell Res Ther. 2018 Apr 6;9:92. doi: 10.1186/s13287-018-0839-8 (PMC5889534; doi:10.1186/s13287-018-0839-8)
Supplement: Supplementary file 5 — Figure S2 showing characterization of iPSC colony 5. a Karyotype of iPSC colony 5 was normal. b qRT-PCR analysis showed expression of OCT4, SOX2, and NANOG of iPSC colony 5. PBMNCs of patient used as negative control, H1 embryonic stem cells used as positive control. c Immunofluorescence staining showed expression of TRA-1-60, SSEA4, OCT4, and NANOG. d Sections of teratomas stained with H&E (endoderm: pancreas; mesoderm: muscle; ectoderm: nerve fibers). All scale bars represent 100 μm. (DOCX 1261 kb) [file 13287_2018_839_MOESM5_ESM.docx]

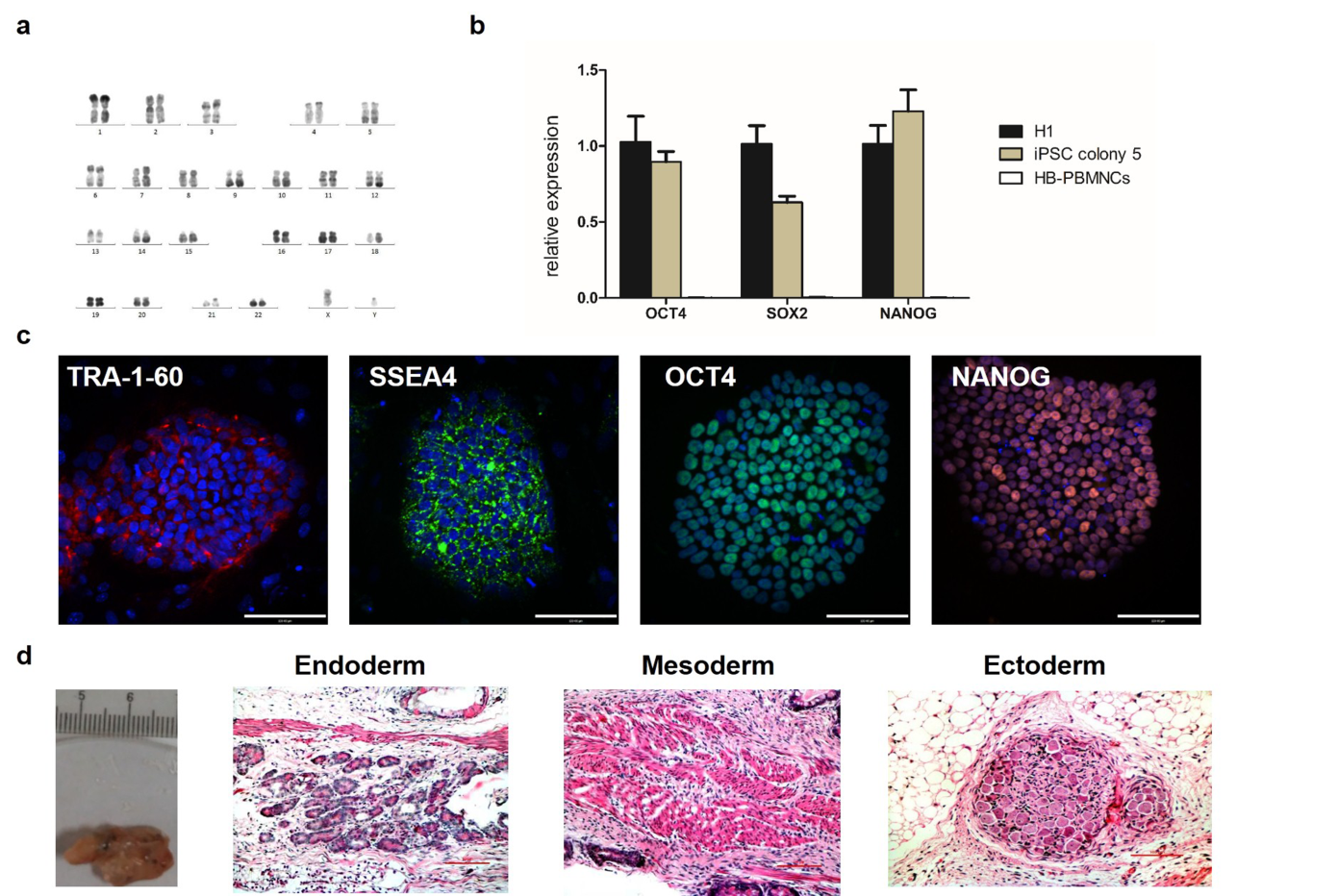


**Additional file 5: Figure S2.** Characterization of iPSC colony 5. All scale bars represent 100 µm. **a.** The karyotype of iPSC colony 5 was normal. **b.** qRT-PCR analysis showed the expression of OCT4, SOX2, and NANOG of iPSC colony 5. The PBMNCs of the patient was used as negative control, while H1 embryonic stem cells were used as positive control. **c.** Immunofluorescence staining showed the expression of TRA-1-60, SSEA4, OCT4, and NANOG. **d.** The sections of teratomas were stained with hematoxylin and eosin (endoderm: pancreas; mesoderm: muscle; ectoderm: nerve fibers.)
