# Supplementary material for: Targeted genome engineering in human induced pluripotent stem cells from patients with hemophilia B using the CRISPR-Cas9 system
Source: Stem Cell Res Ther. 2018 Apr 6;9:92. doi: 10.1186/s13287-018-0839-8 (PMC5889534; doi:10.1186/s13287-018-0839-8)
Supplement: Supplementary file 6 — Figure S3. showing off-target effects detection in successful inserted iPSCs. Using Cas-OFFinder, 1799 potential off-target sites that differed from the sgRNA sequence by up to five nucleotides in the genome were found. We found 97,968 indels, 3084 SVs, 51,628 SNPs, and 2225 CNVs unique to the inserted iPSCs compared to that in the parental iPSCs. Since indels and SVs comprise virtually all of the mutations introduced by CRISPR-Cas9, we focused solely on indels and SVs. Through comparison of potential off-target sites, and indels and SVs unique to the inserted iPSCs, we found no overlapping mutation between them. (DOCX 302 kb) [file 13287_2018_839_MOESM6_ESM.docx]

**
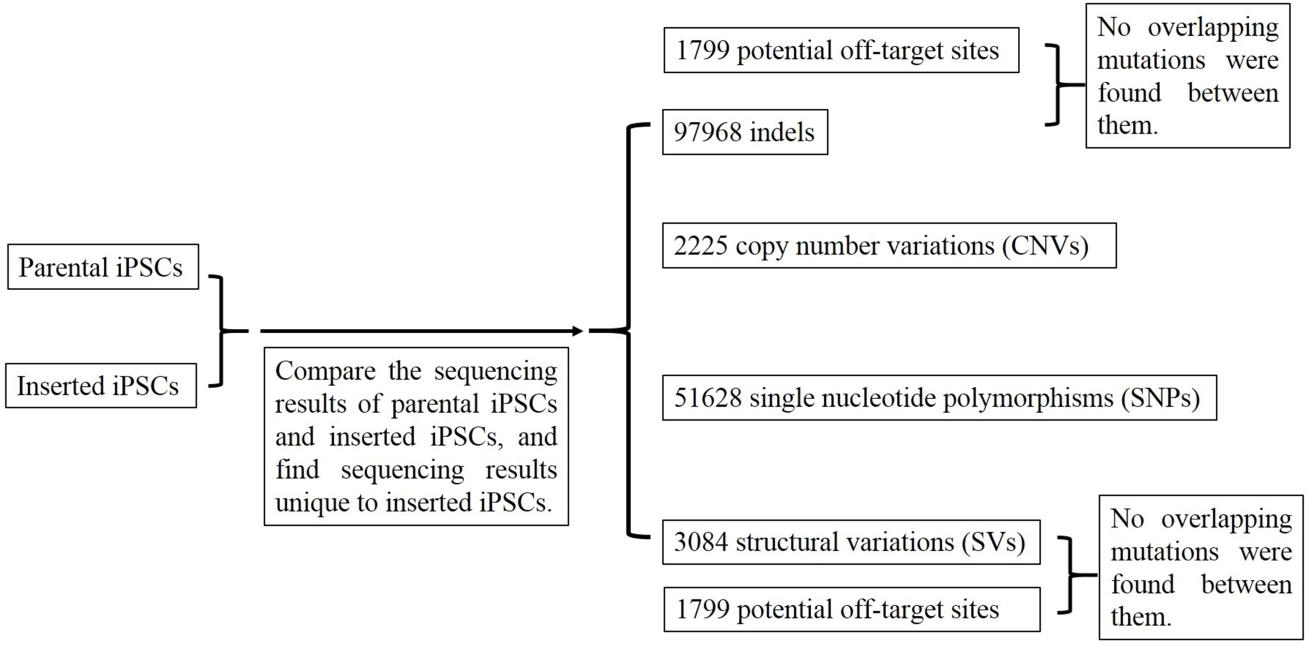
Additional file 6: Figure S3.** Off-target effects detection in the successful inserted iPSCs. Using Cas-OFFinder, 1799 potential off-target sites that differed from the sgRNA sequence by up to five nucleotides in the genome were found. We found 97968 indels, 3084 structural variations (SVs), 51628 single nucleotide polymorphisms (SNPs), and 2225 copy number variations (CNVs) unique to the inserted iPSCs compared to that in the parental iPSCs. Since indels and SVs comprise virtually all of the mutations introduced by CRISPR/Cas9, we focused solely on indels and SVs. Through comparison of potential off-target sites, and indels and SVs unique to the inserted iPSCs, we did not find any overlapping mutation between them.
