# Supplementary material for: Targeted genome engineering in human induced pluripotent stem cells from patients with hemophilia B using the CRISPR-Cas9 system
Source: Stem Cell Res Ther. 2018 Apr 6;9:92. doi: 10.1186/s13287-018-0839-8 (PMC5889534; doi:10.1186/s13287-018-0839-8)
Supplement: Supplementary file 7 — Figure S4. showing characterization of hepatocytic functions. Differentiated cells had functions of glycogen storage (a) and ICG uptake (b), and also expressed LDL-receptor (c) and had ability for LDL uptake (d). All scale bars represent 100 μm. (DOCX 1747 kb) [file 13287_2018_839_MOESM7_ESM.docx]

**
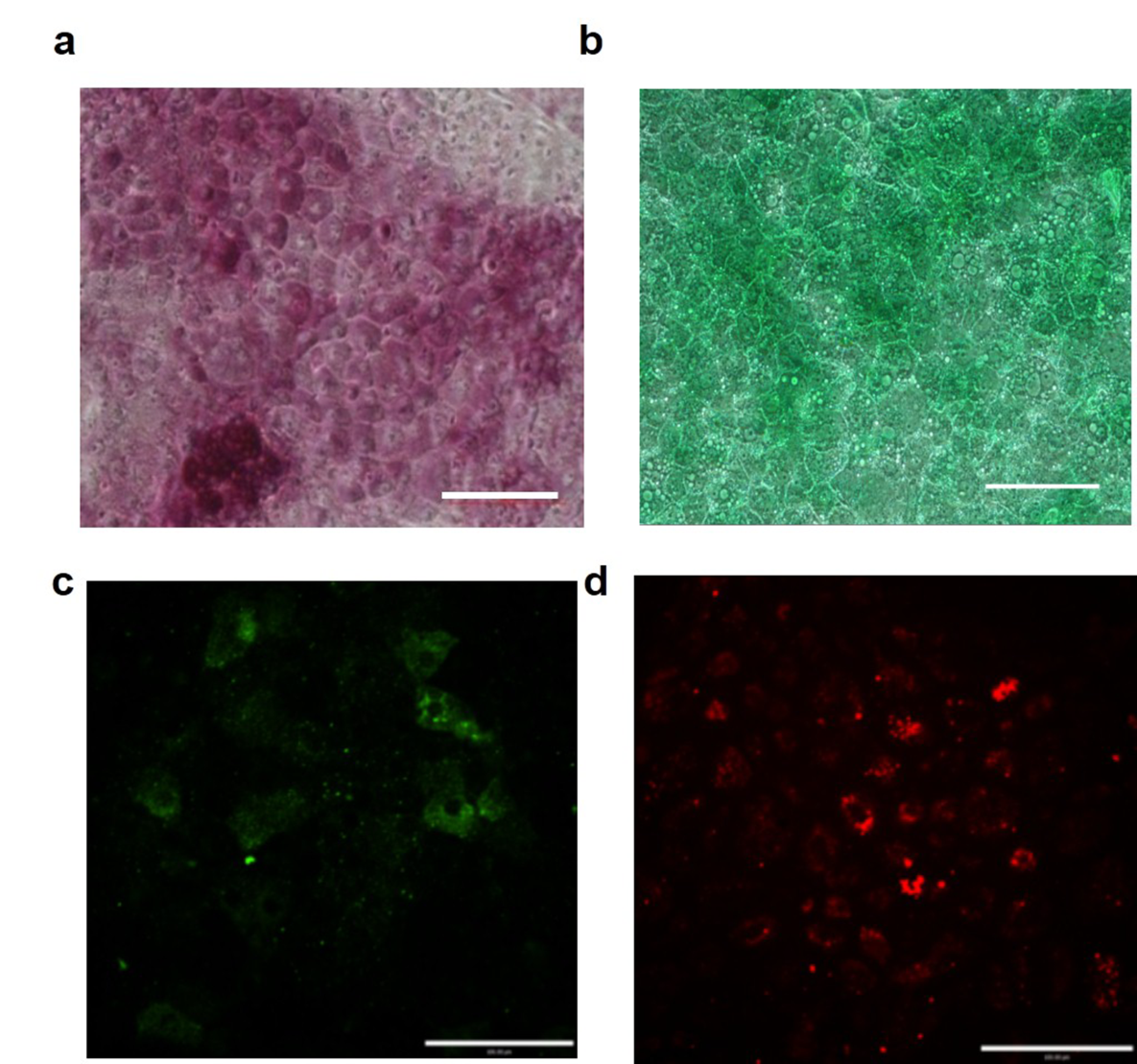
**

**Additional file 7: Figure S4.** Characterization of hepatocytic functions. All scale bars represent 100 µm. The differentiated cells had the functions of glycogen storage (**a**), ICG uptake (**b**). The differentiated cells also expressed LDL-receptor (**c**) and had the ability of LDL uptake (**d**).
